# Cloud-Based Applications for Organizing and Reviewing Plastic Surgery Content

**Published:** 2015-11-09

**Authors:** Anna Luan, Arash Momeni, Gordon K. Lee, Michael G. Galvez

**Affiliations:** Division of Plastic and Reconstructive Surgery, Stanford University Medical Center, Stanford, Calif

**Keywords:** cloud computing, plastic surgery education, technology, mobile applications, surgical training

## Abstract

Cloud-based applications including Box, Dropbox, Google Drive, Evernote, Notability, and Zotero are available for smartphones, tablets, and laptops and have revolutionized the manner in which medical students and surgeons read and utilize plastic surgery literature. Here we provide an overview of the use of Cloud computing in practice and propose an algorithm for organizing the vast amount of plastic surgery literature. Given the incredible amount of data being produced in plastic surgery and other surgical subspecialties, it is prudent for plastic surgeons to lead the process of providing solutions for the efficient organization and effective integration of the ever-increasing data into clinical practice.

## THE COMPLEXITY OF PLASTIC SURGERY

*A thorough familiarity with what has been published in plastic surgery and allied specialties makes available to the surgeon a vast bank of pertinent information gleaned from the experience of many extending far beyond what any one individual could accrue in his entire lifetime*.

Dr D. Ralph Millard, Jr,^1^ made this statement with incredible foresight in 1986. Almost 3 decades later, this statement remains true, particularly in light of the progressive complexity of plastic surgery with increasing overlap with numerous other surgical subspecialties, such as dermatological surgery, oculoplastic surgery, oral-maxillofacial surgery, otolaryngology, and orthopedic surgery.^2^^,^^3^ The increasing amount of knowledge that must be mastered over the course of a 6- to 7-year residency training, followed by lifelong education, can be a daunting task.

## DATA EXPLOSION

**Figure 1:**
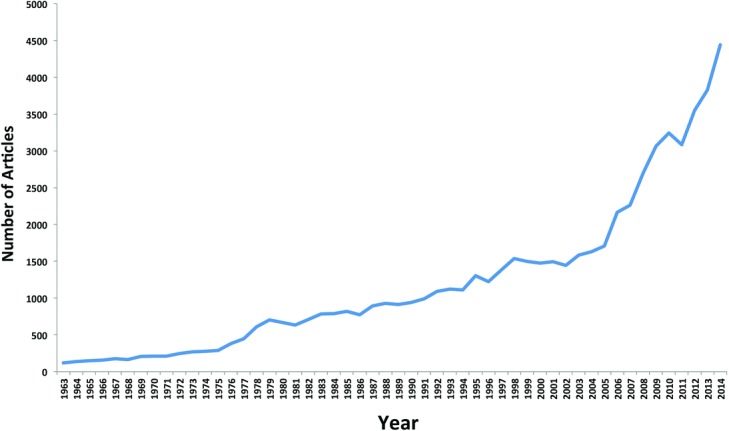
Number of published articles in the following journals from 1963 to 2014: *Aesthetic Surgery Journal*, *Annals of Plastic Surgery*, *Archives of Plastic Surgery*, *Clinics in Plastic Surgery*, *ePlasty, Hand Surgery*, *Journal of Craniofacial Surgery*, *Journal of Hand Surgery* (*American and European*), *Journal of Plastic, Reconstructive & Aesthetic Surgery*, *Plastic* and *Reconstructive Surgery* (*PRS*), and *PRS Global Open* (*GO*).

The number of published articles has increased substantially over the past five decades. In addition, the number of journals publishing content with a focus on plastic surgery has likewise increased in recent years. These journals include *Aesthetic Surgery Journal*, *Annals of Plastic Surgery*, *Archives of Plastic Surgery*, *Clinics in Plastic Surgery*, *ePlasty, Hand Surgery*, *Journal of Craniofacial Surgery*, *Journal of Hand Surgery (American and European)*, *Journal of Plastic*, *Reconstructive & Aesthetic Surgery*, *Plastic and Reconstructive Surgery* (*PRS*), and *PRS Global Open* (*GO*) among others. Figure 1 demonstrates the incredible increase in the number of indexed articles (PubMed) in plastic surgery journals over time. While 119 articles were published in 1963, a total of 4441 articles were indexed in 2014.

Naturally, this increase in content cannot be indexed, reviewed, and implemented into clinical practice without the help of modern technology. In addition to traditional print journals and open access online journals, newer avenues have established themselves as viable means to deliver content. These online-based platforms, such as the Plastic Surgery Education Network, provide useful and up-to-date information that facilitate resident education as well as continued education for board-certified plastic surgeons. Similarly, the Readily Available Digital Aesthetic Resource (RADAR) allows for the customization and annotation of digital medical libraries and is only available to members and subscribers.

While the increase in data is encouraging as it may be a surrogate marker for ongoing progress, review and retention of information become progressively challenging. An example particularly relevant to plastic surgery residents in training is the annual In-Service Examination, which covers all aspects of plastic surgery and draws its content from numerous different sources. In addition to being expanded in content in the upcoming year, a recent study demonstrated that citations from the journals *Annals of Plastic Surgery* and *PRS* merely represent about 4.3% and 38% of all questions, respectively.^4^ In light of these observations, it seems prudent to identify a way with which to integrate content derived from a variety of different sources on a single, easy-to-review platform.

## ACCESSIBILITY OF DATA

**Figure 2:**
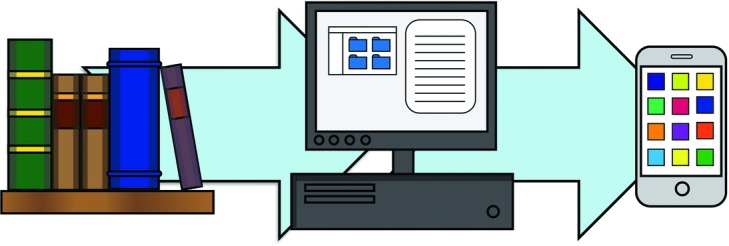
From left to right: the progression of literature found in textbooks, to desktop computers, and now on portable smartphones and tablets.

The traditional approach endorsed by generations of medical students and surgeons to capturing information was by means of taking handwritten notes and sketches. While quick and easy, ubiquitous availability was an issue as these hardcopies could be misplaced forever. Accessibility of data was furthermore a challenge when considering that the sources of information were typically large libraries of books and articles. The contemporary era of digital technology, however, is characterized by the review of medical content via portable smartphones and tablets (Fig 2).^5^^,^^6^ Most textbooks are now released with concurrent digital copies. Stand-alone applications for smartphones and tablets have also been released for specific plastic surgery journals with subscriptions (such as *Annals of Plastic Surgery* and *PRS*), although these are typically limited to a format similar to picking up a journal off the bookshelf (including the product advertisements) and rarely include the ability to annotate articles.

Plastic surgeons are thought to be the first to adopt emerging technologies and therefore can play a leading role in managing this vast amount of content.^7^^,^^8^ A recent study demonstrated the value of integrating a tablet in plastic surgery training, with the possibility of improving clinical efficiency and promoting resident education.^9^ Tablet computer use has further been demonstrated to increase resident access to journal articles, didactic presentations, note-taking programs, and online storage and organization programs.^10^ Other fields such as radiology have incorporated online applications that serve as comprehensive and user-friendly repositories of educational images.^11^ The utility of the smartphone and tablet in a hospital and clinic setting is being increasingly recognized in medicine,^12^^-^17 including their utility in facilitating surgeon-patient interactions.^18^ Certain educational and reference smartphone apps have not only been highlighted in aiding plastic surgeons^19^ but also in particular will change the way that we save and organize the vast amount of plastic surgery literature.

## WHAT ARE CLOUD-BASED APPLICATIONS?

Cloud computing is a model of storage, sharing, and access of data and/or software. Cloud computing involves deploying groups of remote servers and software networks (typically owned and managed by a hosting company) that allow the centralization of data storage and easy online access to computer services or resources. This is in contrast with storing data on personal hard drives, which is referred to as local storage. Cloud-based applications utilize the Cloud to store data online in order to have ease of accessibility, to have data synchronized easily across multiple devices, and to allow for new methods of learning.^20^

Examples of Cloud-based applications include Box (Box Inc; Los Altos, California), Dropbox (Dropbox, Inc; San Francisco, California), Google Drive (Google Inc; Mountain View, California), Evernote (Evernote Corporation; Redwood City, California), Notability (Ginger Labs, Inc.; Palo Alto, California), and Zotero (Roy Rosenzweig Center for History and New Media of George Mason University; Fairfax, Virginia) (Table 1). Notably, the basic application (for smartphones, tablets, laptops, or desktop computers) for each of these programs is free of cost. For an additional fee, the storage space, security, and other features can be upgraded. Notably, there is a paucity of research to date on the use and integration of virtual resources into residency programs and surgical training.^21^^-^23

## ADVANTAGES OF THE CLOUD

The Cloud offers several major advantages that render it highly suitable for surgeons in practice and training. Most obviously, utilization of the Cloud for information storage allows for accessibility of data across multiple different types of devices and virtually at any time, without the need for physical documents (eg, textbooks, printed journals), transfer devices (eg, external hard drives, flash drives), or reliance on any one particular device (eg, a personal laptop computer).^24^ Devices with otherwise limited capabilities, such as smartphones, may be used in conjunction with Cloud software to store, read, and edit resources (with word-processing and spreadsheet software, for example), thereby expanding upon the built-in capabilities of the device. Moreover, it offers enhanced ease of access of specific data. For example, one is able to search for key words in entire sets of separate documents, whether a textbook or research article, and may even include a search for words within figures. Snapshots of portions of articles or PDFs can be integrated together and/or with notes, alleviating the burden of sifting through innumerable separate items. This vastly increases time efficiency and improves the integration of multiple sources of information. Finally, storage on the Cloud provides the means to not only sync across multiple personal devices but also share resources more effectively with others, facilitating knowledge transfer among colleagues and students. These permissions can be given with different privacy levels, varying from read-only to fully editable.

## LIMITATIONS AND DOWNSIDES OF THE CLOUD

The nature of utilizing resources on the Cloud is such that one does not necessarily have the same reliability of reading the content in its entirety compared with when using a physical resource, such as in a journal with highlighting and notes. This likely will change due to applications such as Notability (Ginger Labs, Inc), which allows one to annotate the PDF directly. In addition, access can be limited by the absence of an Internet or other connection for data transfer; however, several programs now offer options for offline content. There also may exist the fear that the Cloud provider may malfunction or otherwise fail with a loss of one's entire digital library; this may be mitigated by performing regular backups of data on another device. Cultural inertia, or resistance to changing habits, may also prove to be a challenge in the adoption of Cloud-based applications.

Security is also of utmost importance. As aspects of patient care become increasingly electronic in nature, measures must be taken to secure any information that is confidential^25^ and also to be conscientious of what is uploaded to the Cloud (Table 2). Naturally and rightfully so, there are concerns to be had regarding the security of Cloud storage.^26^ However, companies such as Box are also partnering with institutions to provide highly secure encrypted versions of storage that allow for the inclusion of sensitive information. As demand increases for secure Cloud computing, we anticipate that this trend will continue in the near future.

Another valid concern is that some material amassed on the Cloud may be copyrighted material (owned by either the journal itself or authors in an open access journal). Infringement of copyright, trademark, or other intellectual property laws is subject to possible legal action by the owner. The several companies that create Cloud applications have terms of service that respect intellectual property and expect its users to do the same. Specifically, these terms of service are in accordance with the Digital Millennium Copyright Act of 1998 (the text can be found on the US Copyright Office Web site).^27^ One may have to obtain permission from the copyright owner, or use may fall within fair use. However, copyright law on fair use is quite complex, with several Web sites dedicated to providing guidelines.^28^^,^^29^ Before sharing a note or document, especially in the public domain, one must make sure that none of the work has copyrighted material. Duplication and sharing of content with others who do not have specific journal access are prohibited and illegal. It is notable, however, that any facts discovered in research are in the public domain and are free to all.

Finally, there is concern that as all these data are amassed and stored as opposed to truly learned by the student or surgeon, this will result only in familiarity of the material (superficial or incomplete knowledge) as opposed to true recollection (richer associations and the ability to explain to others).^30^^,^^31^ The best way to test knowledge is through testing and assessment of what the student or surgeon really knows. This is particularly crucial when measuring progress and defining training outcomes as a trainee progresses from a novice to an expert, especially with the advent of the Plastic Surgery Milestone Project.^32^ As of now, it is not clear whether the Cloud contributes to relying on superficial knowledge or assists with learning and true recollection with application of these data.^33^ However, there are studies in the anesthesia literature to support the benefits of having mobile device applications for just-in-time learning with improvements in confidence, clinical evaluation, and performing procedures.^34^

## CURRENT LIMITATIONS IN THE LITERATURE AND LOCAL STORAGE

Publishing companies unfortunately have different levels of access for users, which are paid either by universities or by personal subscriptions. Open access journals offer content free to any viewer, as well as allow authors to retain their copyright of the materials published. Notably, there are two fully open-sourced plastic surgery journals: *ePlasty* and *PRS GO*. However, once access is obtained to a journal, there continue to be limitations on one's ability to search and locate the desired information. For example, users typically lack the ability to search the inner contents of a manuscript. Instead, when searching in PubMed or a specific journal's search menu online, one is only able to search the title, abstract, and specified key words. The Cloud, however, can serve as a repository of selected articles or content on a topic, rather than having to conduct a comprehensive search on the subject.

In addition, downloaded articles are commonly titled as a garbled number (likely for indexing within the journal's articles), which does not help the clinician to easily store the journal article in an organized manner. Once downloaded and saved, the entire content with the article may then be queried; however, renaming it appropriately will be necessary. We recommend renaming the journal article file in the following format: “[First author]—[Year], [Journal], [Title].” Finally, articles[Fig F3][Fig F4] that have been read in the past can be difficult to relocate unless specific details are remembered, whether through a database or hard drive search.

## RECOMMENDATIONS AND ALGORITHM

**Figure 3 F3:**
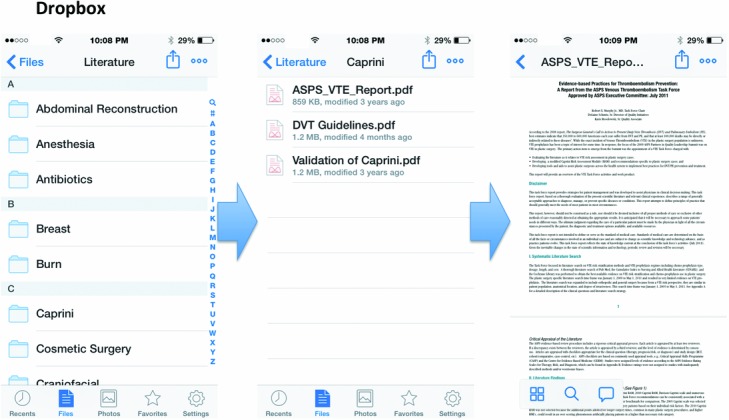
Cloud Document Storage application example with Dropbox, which is utilized to search for a specific topic and documents (eg, DVT prophylaxis). DVT indicates deep vein thrombosis; VTE, venous thromboembolism.

**Figure 4 F4:**
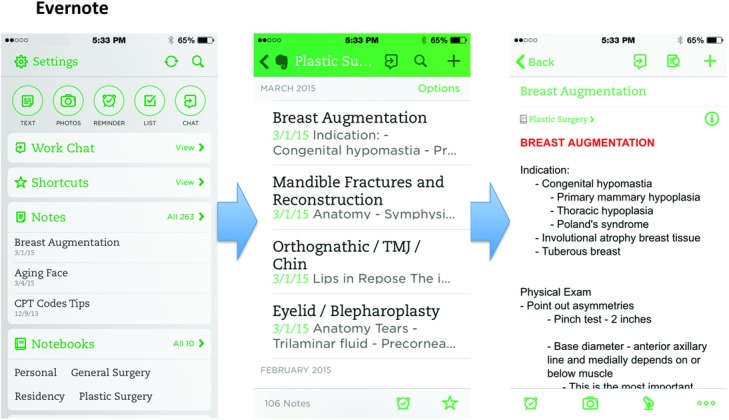
Cloud Note Taking application example with Evernote, which is utilized to store written notes on specific plastic surgery topics (eg, breast augmentation). CPT indicates Current Procedural Terminology; TMJ, temporomandibular joint.

Given the organization required for all these data, here we provide a personal solution, taking into account that every individual surgeon has different learning habits. Every library for each surgeon will be unique. First, we recommend the use of two separate programs by each surgeon, including a Cloud Document Storage program (Fig 3) and a Cloud Note Taking program (Fig 4). On our evaluation of these programs, there does not yet appear to be an existing program that easily integrates the use of both note taking and file management.

**Figure 5:**
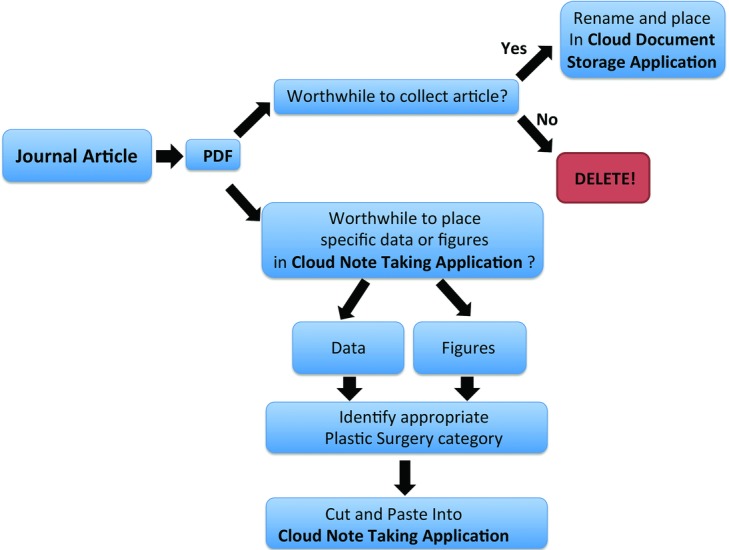
Algorithm for organizing journal articles on the Cloud: reading an article, banking it in a Cloud-based storage, and cutting and pasting relevant information into Cloud applications.

A simple algorithm can demonstrate the effective use of Cloud-based programs in organizing plastic surgery literature (Fig 5). First, upon reading and downloading a PDF of a journal article, the user must first decide whether the article content is useful. If the major portion of the article is valuable, the file should be renamed in a structured manner and saved in a Cloud Document Storage application. Depending on the application used, the file may be placed in a specific pertinent folder/collection, and/or associated with appropriate tags or key words to categorize the document. The user may also annotate or highlight the file as desired. If portions of the article (eg, a particular figure or specific data) are deemed to be worthwhile to save, they can be cut and pasted selectively into a Cloud Note Taking application under the appropriate category with other related information.

There will need to be further discussion on innovative ways on how to organize this extensive amount of literature in the field. While the use of mobile devices has rapidly expanded and there is a strong desire for the integration of mobile computing technology into clinical practice and education, adoption of mobile and Cloud-based applications is still lacking overall.^6^^,^^35^ However, current Cloud-based applications have become increasingly sophisticated in recent years. Available Cloud platforms now offer highly useful methods of not only interfacing with traditional formats of learning, such as textbooks, but also enhancing the ability to utilize their content effectively. The stage is now set for improved integration into the clinical setting, and we must learn how to best use these new digital tools in the plastic surgeon's armamentarium.

## CONCLUSION

Cloud-based applications have the potential to maximize the efficiency and productivity of surgeons in practice and training. As Cloud computing becomes increasingly commonplace, we must learn how to integrate it into our professional lives in a manner that is effective and comprehensive while secure. Particular use of these applications may include the organization of literature and the sharing of data. Our proposed algorithm incorporates Cloud-based applications to save and organize the vast amount of plastic surgery literature in hopes of rapid clinical reference and use. To our knowledge, this is the first article to highlight the utility of Cloud-based applications in plastic surgery.

## Figures and Tables

**Table 1:** Available platforms for working with plastic surgery content in the Cloud

**Evernote (evernote.com)**
**Primary Utility:**
Note Taking
**Description:**
Platform for note taking, allowing for clipping of web articles, photos, handwritten notes, etc. Collaboration with other users.
**Cost:**
Free (Basic), $24.99/year (Plus), $49.99/year (Premium)
**Compatible Devices:**
Apple, Android, Windows, BlackBerry, Amazon
**Unique Features:**
Evernote Plus: Access to notes offline, directly saving e-mails, passcode lock to access app, monthly uploads up to 1GB.
Evernote Premium: Unlimited uploads, conversion of notes to presentation format, annotate PDFs, search in documents and attachments, digitize business cards. (In addition to Plus features).
**Notability (gingerlabs.com)**
**Primary Utility:**
Note Taking
**Description:**
App for note taking, incorporating text-based notes, handwriting, drawings, images, embedded documents or web pages. Allows annotation of other imported files (e.g. PDFs). Notes may be saved in iCloud or exported into other formats for sharing via app or Dropbox.
**Cost:**
$2.99 (Mobile app), $5.99 (Desktop app)
**Compatible Devices:**
Apple (iPhone, iPad, iPod touch, Mac desktop)
**Unique Features:**
Syncs audio recording with note, creating bookmarks.
**Box (box.com)**
**Primary Utility:**
Document Storage
**Description:**
Platform for document/photo/video syncing across devices, document editing, organization, searching, and secure sharing. Partnered with more than 1,000 other apps (OneCloud) for additional utilities
**Cost:**
Free (Personal), $5/user/month (Starter), $15/user/month (Business), Variable (Enterprise)
**Compatible Devices:**
Apple (iPhone, iPad), Android, Windows Phone, BlackBerry, Mobile website
**Unique Features:**
Personal: 10 GB storage, 250 MB file upload size. Microsoft Office integration. Offline access. Two-factor authentication.
Starter: Shared workspace with 3–10 users, 100 GB storage, 2 GB file size. Access permissions include auto-expiration, file locking, and history. (In addition to Personal features).
Business: Minimum 3 users, unlimited storage, 5 GB file size. Additional mobile security controls. (In addition to Starter features).
**Dropbox (dropbox.com)**
**Primary Utility:**
Document Storage
**Description:**
Platform for storage and sharing of documents, photos, videos, and other files. Syncs across all devices and Dropbox website. Allows sharing to files or folders.
**Cost:**
Free (Basic), $9.99/month (Pro), $15/user/month (Business)
**Compatible Devices:**
Apple (iPhone, iPad), Android, Windows (Phone, Tablet), BlackBerry, Kindle Fire, Mobile website
**Unique Features:**
Basic: 2 GB storage.
Pro: 1 TB storage. Additional sharing controls. Remote wipe.
Business: Unlimited storage, unlimited file recovery, file sharing controls.
**Google Drive (drive.google.com)**
**Primary Utility:**
Document Storage
**Description:**
Cloud storage and editing of photos, videos, presentations, PDFs, Microsoft Office files. Sharing capabilities include viewing, commenting, and editing. Encrypted with SSL. Files can be available offline.
**Cost:**
Free (Default, 15 GB storage), $1.99/month (100 GB), $9.99/month (1 TB)
**Compatible Devices:**
Apple (iPhone, iPad, Mac desktop), Android, PC
**Unique Features:**
Can automatically save any Gmail attachment to Drive. Search recognizes objects in images and text in scanned documents. Google Docs, Sheets, and Slides apps within Drive allow making a text document, spreadsheet, or presentation within the app itself. Google Forms allows creation and distribution of online forms or surveys, with export of data into a spreadsheet. Google Drawings allows diagrams/flow charts with incorporation into other documents. Old versions of documents (up to 30 days) available to revert to previous versions or track changes.
**Zotero (zotero.org)**
**Primary Utility:**
Document Storage
**Description:**
Research tool that allows storage of research articles (PDFs, images, audio/video, snapshots of web pages) in a searchable personal library. Automatically synchronized across devices and available online. Groups may be created to share and collaborate on research libraries.
**Cost:**
Free (Default, 300 MB), $20/year (2 GB), $60/year (6 GB), $120/year (Unlimited)
**Compatible Devices:**
Mac, Windows, Linux, Mobile website. No app but multiple third-party applications exist.
**Unique Features:**
Automatically indexes content of the personal library, and organizes content into customized collections and subcollections. Tags may be assigned to library items to organize via keywords that may be used to filter items later. Duplicates may be detected and merged. Notes can be stored in the library either as an attachment to an item or as a standalone note. Citations may be created directly in word processing software, emails, or Google documents by dragging references from Zotero.

**Table 2:** Recommendations of information to include and exclude in a note taking application*

Include	Exclude
Notes by plastic surgery subject (eg, breast reconstruction, craniofacial surgery, aesthetic surgery, hand surgery, lower extremity reconstruction, burn)	Protected Health Information (eg, patient names, MRNs, contact information)
Surgery indications and operative plan notes/diagrams	Patient photographs
Specific case information (without patient identifiers)	Personal information (eg, passwords, credit cards)
Figures and images of relevant anatomy for surgical procedure	Copyrighted material
Patient education notes	
Billing information and coding	
Medical management notes (e.g. DVT prophylaxis)	
Pharmacology and toxicology	
Research projects	
Operating room and surgeon-specific preferences	
Hospital-specific notes (locations, call schedules, etc)	

*DVT indicates deep vein thrombosis; MRN, medical record number.
